# Numerical and Experimental Evaluation of Error Estimation for Two-Way Ranging Methods †

**DOI:** 10.3390/s19030616

**Published:** 2019-02-01

**Authors:** Cung Lian Sang, Michael Adams, Timm Hörmann, Marc Hesse, Mario Porrmann, Ulrich Rückert

**Affiliations:** Cognitronics and Sensor Systems Group (CITEC), Bielefeld University, 33619 Bielefeld, Germany; madams@techfak.uni-bielefeld.de (M.A.); thoerman@techfak.uni-bielefeld.de (T.H.); mhesse@techfak.uni-bielefeld.de (M.H.); mporrman@techfak.uni-bielefeld.de (M.P.); rueckert@techfak.uni-bielefeld.de (U.R.)

**Keywords:** TEEM, TWR, AltDS-TWR, SDS-TWR, distance measurement, error analysis, delay effects, TOF error model

## Abstract

The Two-Way Ranging (TWR) method is commonly used for measuring the distance between two wireless transceiver nodes, especially when clock synchronization between the two nodes is not available. For modeling the time-of-flight (TOF) error between two wireless transceiver nodes in TWR, the existing error model, described in the IEEE 802.15.4-2011 standard, is solely based on clock drift. However, it is inadequate for in-depth comparative analysis between different TWR methods. In this paper, we propose a novel TOF Error Estimation Model (TEEM) for TWR methods. Using the proposed model, we evaluate the comparative analysis between different TWR methods. The analytical results were validated with both numerical simulation and experimental results. Moreover, we demonstrate the pitfalls of the symmetric double-sided TWR (SDS-TWR) method, which is the most highlighted TWR method in the literature because of its highly accurate performance on clock-drift error reduction when reply times are symmetric. We argue that alternative double-sided TWR (AltDS-TWR) outperforms SDS-TWR. The argument was verified with both numerical simulation and experimental evaluation results.

## 1. Introduction

The field of localization systems in wireless communications is growing since it enables a wireless mobile node to have both data communication and positioning capabilities. The localization process is typically categorized into two phases: (i) ranging (measurement) phase, during which the distance between the transceivers is measured, and (ii) positioning (location-update) phase, during which the current position of the wireless node is determined using the knowledge from the ranging phase and positioning algorithms [1]. Regarding positioning, besides wireless-only positioning systems, multiple sensor approaches, like diversity navigation, have been proposed as well [1]. In those systems, ranging is supported by using additional information, e.g., from an Inertial Measurement Unit (IMU). In this paper, we focus on the accuracy of wireless ranging based on Ultrawide Bandwidth (UWB), and specifically study different Two-Way Ranging (TWR) methods available in the literature.

TWR plays an important role in measuring the distance between two wireless transceiver devices when clock synchronization is not available or absent in a time-based localization system. By knowing the Time of Flight (TOF) between the two transceivers, i.e., a signal’s traveling time in free space, the distance between them can easily be measured using the speed of light. However, it is necessary that the two transceivers have a synchronized clock (same clock domain) in such one-way ranging systems.

In the TWR approach, a set of time periods (e.g., $t_{round}=10\;\mu s$ and $t_{reply}=4\;\mu s$) is used to calculate the distance between two transceivers (Section 2) instead of using direct timestamps. This is because the period of a certain time is the same for every device regardless of their own clock references. However, because of the imperfections of clock oscillators in the real physical world, a clock drifts away even if it is perfectly tuned in the initial state [2]. These clock drifts cause inaccuracy in measuring the mentioned time periods, especially when the application requires centimeter-level accuracy. This is because 1 ns of TOF error can lead to an approximate error of 30 cm in distance estimation [3]. For this reason, there are several TWR methods available in the literature to minimize this inaccuracy in ranging due to clock drifts (Section 2).

As a consequence, the existing TOF error-estimation model for TWR, described in the IEEE 802.15.4-2011 standard, tackles clock drifts as the only dominant errors [3] (pp. 258–275). However, this model is inadequate for analysis of system performance between different TWR methods, especially when it is important to identify a better method for a certain application. For instance, the performance difference between two closely related TWRs, such as a symmetric double-sided TWR (SDS-TWR) and alternative double-sided TWR (AltDS-TWR), cannot be definitely clarified using the existing model [4]. Moreover, AltDS-TWR is robust against the variation of reply time, as we discuss in Section 7.3.1, which cannot be explained with a conventional clock-drift model, as above.

In this paper, we propose a novel Time-of-Flight Error Estimation Model (TEEM) for TWR methods, which is an extended version of the IEEE 802.15.4-2011 standard [3] (pp. 258–275). Regarding this, a delay in message delivery (Section 3.1) is accounted as a feature in the proposed model. In fact, this delay is crucial and fundamental, because TOF error is affected not only by clock drift in the oscillator but also by other error sources, such as propagation time delay [5], transmission time delay, and receiving time delay [2]. That includes the delay introduced by the antenna, PCB, and other external and internal electronic components.

In addition, we demonstrate the pitfalls of the most highlighted TWR techniques in the literature, namely, SDS-TWR. Conventionally, SDS-TWR is commonly used to illustrate the reduction of TOF error due to clock drifts in wireless ranging systems [3]. Concerning this, we argue that AltDS-TWR is more robust than SDS-TWR in all aspects.

This article is the extended version of our previous conference paper, presented in IPIN 2018 [6]. Three significant changes were made. Firstly, experiment results for different TWR methods are given to validate the simulation results presented in the conference paper (Section 7). Secondly, we provide the generic delay model for TWR methods (Section 3.1 and Section 3.2), which was regarded as a propagation time-delay error in our previous work [6]. Thirdly, we verify our argument, which is that AltDS-TWR method outperforms SDS-TWR, with both numerical simulation (Section 6) and experimental evaluation (Section 7) results.

This paper is organized as follows: In Section 2, the overview of TWR methods, the existing standard TOF error-estimation approach, and related work are addressed. Then, the foundation of the proposed TOF error-estimation model is established in Section 3, followed by analytical comparison between the proposed and conventional TOF error estimation in Section 4. A comparative study between four TWR methods using the proposed model is provided in Section 5, and the numerical simulation results are presented in Section 6. Then, the experimental evaluation results are given in Section 7, and a summarized discussion in Section 8. Final conclusions are drawn in Section 9.

## 2. State of the Art for TWR Methods and Related Work

In this section, we address four commonly used TWR methods in time-based wireless localization systems and the existing TOF error-estimation model, given in the IEEE 802.15.4-2011 standard. IEEE 802.15.4 uses clock drifts as the only dominant error to compare TOF errors among different TWR methods [3].

Brief introductions for each of the evaluated TWR methods, which are the single-sided TWR (SS-TWR), (symmetric) DS-TWR, AltDS-TWR, and asymmetric double-sided TWR (ADS-TWR), are presented in this section. These methods were carefully chosen to reflect the general overview of the available TWR methods in the literature. The remaining TWR methods, derived mainly from the presented techniques, are: SDS-TWR with multiple acknowledgments [7], asynchronous double TWR (D-TWR) [8], burst-mode SDS-TWR [9], SDS-TWR with unequal reply-time method [10], TWR using estimated frequency offsets [11], parallel DS-TWR [12], and passive extended DS-TWR [13].

Apart from measuring distances between transceivers in wireless communications, TWR has also been widely applied in networkwide clock-synchronization algorithms for wireless sensor networks (WSN) [14,15,16,17].

### 2.1. (Simple) SS-TWR

**Figure 1:**
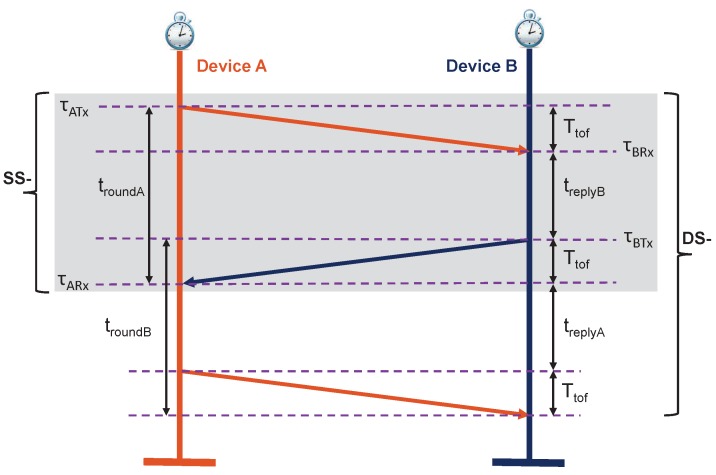
Illustration of single- and double-sided Two-Way Ranging (TWR) methods (©2018 IEEE. Reprinted with permission).

For SS-TWR [3,18] (the shaded area in Figure 1), the round-trip time of the signal can be formulated as:

(1) $$t_{roundA}=2\;T_{tof}+t_{replyB}$$

where $t_{roundA}=\tau_{ARx}-\tau_{ATx}$ is the true round-trip time of a signal measured at Device A and $t_{replyB}=\tau_{BTx}-\tau_{BRx}$ is the true reply time of a signal measured at Device B (Figure 1). $\tau_{ATx}$ and $\tau_{ARx}$ are the transmitted and received timestamps measured at Device A, and $\tau_{BTx}$ and $\tau_{BRx}$ are the transmitted and received timestamps measured at Device B, respectively.

In particular, the round-trip time of a signal ($t_{roundA}$) is measured from the beginning of Device A transmitting the ranging message ($\tau_{ATx}$ in Figure 1) until the reception of the replied signal back from Device B ($\tau_{ARx}$ in Figure 1). Therefore, the TOF for the SS-TWR method can be obtained as:

(2) $$T_{tof}=\frac{1}{2}\left(t_{roundA}-t_{replyB}\right)$$

### 2.2. SDS-TWR

The round-trip time of double-sided TWR [3,18] (Figure 1) can be formulated as:

(3a) $$t_{roundA}=2\;T_{tof}+t_{replyB}$$

(3b) $$t_{roundB}=2\;T_{tof}+t_{replyA}$$

where $t_{roundA}$ and $t_{roundB}$ are the true round-trip times of a signal measured at Device A and B, respectively. $t_{replyA}$ and $t_{replyB}$ are the true reply times measured at Device A and B, respectively.

By combining Equations (3a) and (3b), the resulting TOF for SDS-TWR or DS-TWR can be expressed as:

(4) $$T_{tof}=\frac{1}{4}\left(\left(t_{roundA}-t_{replyA}\right)+\left(t_{roundB}-t_{replyB}\right)\right)$$

In DS-TWR, the ranging time for a single measurement is approximately less than twice as long as SS-TWR due to the additional reply time, as depicted in Figure 1.

### 2.3. AltDS-TWR

The AltDS-TWR method [4] shares the same core concept as Equations (3a) and (3b) from Section 2.2 (Figure 1), as follows:

(5a) $$t_{roundA}=2\;T_{tof}+t_{replyB}$$

(5b) $$t_{roundB}=2\;T_{tof}+t_{replyA}$$

However, instead of combining the two equations, the AltDS-TWR method is achieved by multiplying Equations (5a) and (5b) as:

$$t_{roundA}\;\cdot \;t_{roundB}=\left(2\;T_{tof}+t_{replyB}\right)\;\cdot \;\left(2\;T_{tof}+t_{replyA}\right)$$

By simplifying the equation, the $T_{tof}$ is obtained as follows:

(6) $$T_{tof}=\frac{t_{roundA}\;\cdot \;t_{roundB}-t_{replyA}\;\cdot \;t_{replyB}}{t_{roundA}+t_{replyA}+t_{roundB}+t_{replyB}}$$

The detailed derivation of the formula can be found in Reference [4].

### 2.4. ADS-TWR

**Figure 2:**
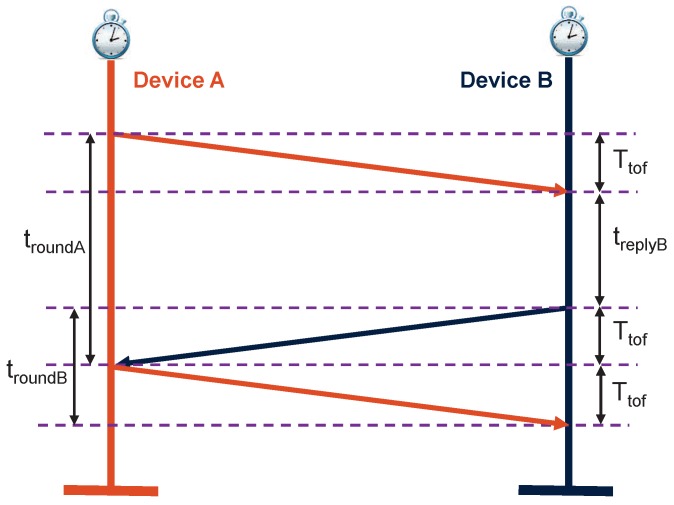
Illustration of the asymmetric double-sided TWR method (©2018 IEEE. Reprinted with permission).

Asymmetric double-sided TWR [19] (Figure 2) can be formulated as follows:

(7a) $$t_{roundA}=2\;T_{tof}+t_{replyB}$$

(7b) $$t_{roundB}=2\;T_{tof}$$

By combining Equations (7a) and (7b), the $T_{tof}$ for ADS-TWR can be achieved as:

(8) $$T_{tof}=\frac{1}{4}\left(t_{roundA}+t_{roundB}-t_{replyB}\right)$$

The major motivation behind the implementation of ADS-TWR is to reduce the ranging time of the system while attaining the same performance level as SDS-TWR or AltDS-TWR.

### 2.5. Conventional TOF Error Estimation Approaches

The existing conventional TOF error-estimation approach, i.e., the IEEE 802.15.4-2011 standard [3] (pp. 258–275), is specifically only based on clock-drift error effects in TWR methods. The fundamental model can be simplified as in the following equations according to the method originally proposed in Reference [18] and presented in Reference [3]. Then, the method was later extensively applied and studied in References [4,8,9,19,20]. The corresponding concept is depicted in Figure 1. The representation of the equations is inspired by the work in Reference [4].

(9a) $${\hat{t}}_{roundA}=\left(1+e_{A}\right)t_{roundA}$$

(9b) $${\hat{t}}_{replyA}=\left(1+e_{A}\right)t_{replyA}$$

(9c) $${\hat{t}}_{roundB}=\left(1+e_{B}\right)t_{roundB}$$

(9d) $${\hat{t}}_{replyB}=\left(1+e_{B}\right)t_{replyB}$$

where ${\hat{t}}_{roundA}$ and ${\hat{t}}_{roundB}$ are the estimated round-trip times of Devices A and B, respectively. $t_{roundA}$ and $t_{roundB}$ are the true round-trip times of Devices A and B, respectively. ${\hat{t}}_{replyA}$ and ${\hat{t}}_{replyB}$ are the estimated replied times of Devices A and B, respectively. $t_{replyA}$ and $t_{replyB}$ are the true replied times of Devices A and B, respectively. $e_{A}$ and $e_{B}$ are the clock-drift errors introduced by Devices A and B, respectively. It is conventionally assumed that $T_{tof}<<t_{replyA}\;\mathrm{or}\;t_{replyB}$. The reason is that reply times are in the order of several milliseconds, while $T_{tof}$ is in the order of nanoseconds [3].

Moreover, a linear algebra approach on error analysis of a co-operative position system using GPS and TWR was performed in Reference [21]. The overall concept is interesting because the presented method can be used as a transition system that bridges the localization systems of UWB (indoor) and GPS (outdoor). However, error analysis performed for TWR in the work is too shallow. The authors assumed in their work that, firstly, clock-drift errors are compensated just by using the SDS-TWR method, and secondly, ranging measurement error is purely white Gaussian noises. This assumption is too broad to reflect the actual TOF error in the TWR method. In addition, the error model and protocol specifically for the parallel double-sided TWR (PDS-TWR) method were performed in Reference [12]. The authors clearly sketch the source of error in two phases, namely, the ranging and localization processes, and focused on the former phase. Then, the variation of ranging error upon symmetric and quasisymmetric cases are discussed. It was proven in their work that PDS-TWR outperforms SDS-TWR. However, the error term used in their proposed model is unclear, which is defined as the difference between a duration measured with the PHY of a node and real duration (ppm). In addition, the presented error model is not generic and defined only for PDS-TWR method.

## 3. Proposed Analytical Model

In the following, we outline the problem statement and sketch various error sources (Section 3.1). Subsequently, we describe our extended error model (Section 3.2).

### 3.1. Problem Statement

TWR methods are excellent in ranging distances between two wireless transceiver devices without using clock synchronization. However, clock-drift errors in oscillators (e.g., ±20 ppm in the IEEE 802.51.4-2011 standard [3]) degrade their performance. The conventional TOF error approach specifically tackles clock drifts as the only dominant error source in TWR methods.

However, the estimation of $T_{tof}$ in a time-based wireless communication system is fundamentally perturbed by various delay error sources as already mentioned in Section 1. These delay sources, especially for time-based localization schemes, can be categorized as follows:

- Propagation-Time Delay (PTD): propagation time is the time required for a message to be transmitted from the transmitter to the receiver in a wireless channel [2]. PTD occurs in two cases: When the direct path signal is completely obstructed or blocked, or when the signal has to traverse through different materials [5]. In other words, PTD occurs when the path of the signal has been reflected or obstructed by obstacles.
- Transmission-Time Delay (TTD): This is the delay caused by the time required for building a message at the application layer (software), accessing time in the medium access control (MAC) layer (protocol), and transmitting time of the message in the physical (PHY) layer [2,14]. This includes delays introduced by the antenna, PCB, and other external and internal electronic components.
- Receiving-Time Delay (RTD): The delay caused by the time required for receiving a message at the PHY, MAC, and application layers, similar to transmission-time delay [2,14].
- Preamble Accumulation-Time Delay (PATD: This is the time required for detecting a certain preamble sequence and finding the start-frame delimiter (SFD) sequence in the PHY layer [22], especially when a coherent receiver [23] is used in the system. PATD is influenced by the presence of a multipath [24] and quick frame arrival time [3] (pp. 261–263) because of a relatively short distance measurement [22] (p. 32). It is more significant when the reflected signal arrives within the chip period of the first path signal [24].

For the sake of simplicity without loss of generality, the mentioned delay errors for $T_{tof}$ estimation in wireless communication systems can be modeled as a simple linear equation. For a single round-trip time in the SS-TWR technique (Figure 1), the total round-trip time delay can be formulated as:

(10) $$\begin{array}{ll} \Delta_{ABA} & =\sum_{i=1}^{n}\left({\mathrm{AB}\_\mathrm{Delay}}_{i}+{\mathrm{BA}\_\mathrm{Delay}}_{i}\right)\\ & \approx 2\;\cdot \;\sum_{i=1}^{n}{\mathrm{Delay}}_{i}\\ & \approx 2\;\cdot \;(\mathrm{TTD}+\mathrm{PTD}+\mathrm{PATD}+\mathrm{RTD}) \end{array}$$

where $\Delta_{ABA}$ is the total delay that occurred within a single round-trip-time of a signal in the TWR method measured at Device A (Figure 1). That is, the total delay produced by a signal transmitted from Device A to B and back to Device A. The Delay can be one or more of the previously mentioned individual delays, which are TTD, PTD, PATD, and RTD. The total number of delays that could affect the mentioned round-trip delay error in the SS-TWR method is given as *n* (10). Note that the constant “2” in Equation (10) appears to represent the two-way traveling routes of a signal in the SS-TWR method for a single measurement. Here, it is assumed that the delays produced in the first route (Device A to B) and the second route (Device B to A) are the same.

Regarding this, the absolute error and relative error for the above-mentioned total delay in the single round-trip-time of TWR (shaded area in Figure 1) can be calculated as follows [25] (p. 62):

(11) $$\epsilon =\mathrm{estimated}\;\mathrm{value}-\mathrm{exact}\;\mathrm{value}={\hat{t}}_{roundA}-t_{roundA}$$

(12) $$\xi =\frac{\mathrm{absolute}\;\mathrm{error}}{\mathrm{exact}\;\mathrm{value}}=\frac{\epsilon }{t_{roundA}}=\frac{{\hat{t}}_{roundA}-t_{roundA}}{t_{roundA}}$$

where ϵ and ξ are the absolute error and relative error of the above-mentioned delay ($\Delta_{ABA}$).

Assuming that the absolute error is only affected by the above-mentioned delay ($\Delta_{ABA}$) in the measurement, the estimated round-trip-time for SS-TWR becomes ${\hat{t}}_{roundA}$$=t_{roundA}+\Delta_{ABA}$. By substituting this value into Equation (12), the relative error for the total delay within the single-round-trip time of SS-TWR can be represented as:

(13) $$\xi_{ABA}=\frac{\Delta_{ABA}}{t_{roundA}}$$

where $\xi_{ABA}$ is the relative error of the total delay in a single-round-trip time of a signal in SS-TWR method measured at Device A (shaded area in Figure 1).

If there is absolutely no delay ($\Delta_{ABA}=0$) between the two transceivers in the SS-TWR method, the corresponding relative error upon round-trip time delay ($\xi_{ABA}$) equals zero. Otherwise, the round-trip time delay ($\xi_{ABA}$) is the relative error achieved from the summation of all related delays along the path. Correspondingly, relative delay errors for the DS-TWR method are $\xi_{BAB}$ and $\xi_{ABA}$.

### 3.2. Proposed TOF Error-Estimation Model

As it is explained in Section 3.1, our proposed model is based on both clock-drift error and the relative error in a round-trip time delay. The analytical formulas for the proposed TOF error-estimation model are provided as follows, in reference to Figure 1:

(14a) $${\hat{t}}_{roundA}=\left(1+e_{A}+\xi_{ABA}\right)t_{roundA}$$

(14b) $${\hat{t}}_{replyA}=\left(1+e_{A}\right)t_{replyA}$$

(14c) $${\hat{t}}_{roundB}=\left(1+e_{B}+\xi_{BAB}\right)t_{roundB}$$

(14d) $${\hat{t}}_{replyB}=\left(1+e_{B}\right)t_{replyB}$$

where $\xi_{ABA}$ and $\xi_{BAB}$ (as introduced in Section 3.1) represent the delay defined as the relative error in the single round-trip time of a signal measured at Device A or B respectively.

Since $\xi_{ABA}$ and $\xi_{BAB}$ represent the relative error of the total delay within a single round trip of a TWR system, it is sufficient that their effects are represented in the estimated round-trip time (${\hat{t}}_{roundA}$ Equation (14a) and ${\hat{t}}_{roundB}$) alone as provided in Equations (14a) and (14c). Therefore, the estimated reply time (${\hat{t}}_{replyA}$ and ${\hat{t}}_{replyB}$) can stay unchanged as in the conventional clock-drift error approach (Section 2.5).

It should be noted that $\xi_{ABA}$ and $\xi_{BAB}$ in Equations (14a) and (14c), defined in Section 3.1, are completely different parameters from clock-drift errors $e_{A}$ and $e_{B}$, which are susceptible to the finite crystal tolerance of the clock oscillators [3].

## 4. Extended State-of-the-Art TWR Methods

In this section, we compare the proposed and conventional TOF error-estimation models on the evaluated four TWR methods.

### 4.1. Extended SS-TWR Method

By using Equation (2), the estimated TOF for the SS-TWR method can be written as:

$${\hat{T}}_{tof}=\frac{1}{2}\left({\hat{t}}_{roundA}-{\hat{t}}_{replyB}\right)$$

where ${\hat{T}}_{tof}$ is the estimated TOF in the system.

The difference between the estimated and true TOF for SS-TWR is:

$${\hat{T}}_{tof}-T_{tof}=\frac{\left({\hat{t}}_{roundA}-{\hat{t}}_{replyB}\right)}{2}-\frac{\left(t_{roundA}-t_{replyB}\right)}{2}$$

By applying Equations (14a) and (14d), the equation becomes:

$${\hat{T}}_{tof}-T_{tof}=\frac{1}{2}\left[\left(e_{A}+\xi_{ABA}\right)t_{roundA}-e_{B}t_{replyB}\right]$$

Substituting $t_{roundA}$ with Equation (1) yields:

$${\hat{T}}_{tof}-T_{tof}=\frac{1}{2}\left[2T_{tof}\left(e_{A}+\xi_{ABA}\right)+\left(e_{A}-e_{B}+\xi_{ABA}\right)t_{replyB}\right]$$

This leads to the TOF error for SS-TWR as:

(15) $${\hat{T}}_{tof}-T_{tof}=T_{tof}\left(e_{A}+\xi_{ABA}\right)+\frac{1}{2}\left(e_{A}-e_{B}\right)t_{replyB}+\frac{1}{2}\xi_{ABA}t_{replyB}$$

For the sake of comparison, the TOF error for SS-TWR using the conventional approach from Equations (9a) and (9d) is:

(16) $${\hat{T}}_{tof}-T_{tof}=T_{tof}e_{A}+\frac{1}{2}\left(e_{A}-e_{B}\right)t_{replyB}$$

It should be noted that our model Equation (15) reduces to conventional Model Equation(16), if $\xi_{ABA}=0$.

### 4.2. Extended SDS-TWR Method

Similar to Section 4.1, if Equation (4) is applied in the proposed error model from Equations (14a)–(14d), and by replacing $t_{roundA}$ and $t_{roundB}$ with Equations (3a) and (3b), the TOF error between the estimated and the true value for SDS-TWR becomes:

(17) $$\begin{array}{ll} {\hat{T}}_{tof}-T_{tof}= & \frac{1}{2}T_{tof}\left(e_{A}+e_{B}+\xi_{BAB}+\xi_{ABA}\right)+\frac{1}{4}\left(e_{A}-e_{B}\right)\left(t_{replyB}-t_{replyA}\right)\\ & +\frac{1}{4}\left(\xi_{BAB}t_{replyA}+\xi_{ABA}t_{replyB}\right) \end{array}$$

For the sake of comparison, the conventional model for TOF error in the SDS-TWR method using Equations (9a)–(9d) is:

(18) $${\hat{T}}_{tof}-T_{tof}=\frac{1}{2}T_{tof}\left(e_{A}+e_{B}\right)+\frac{1}{4}\left(e_{A}-e_{B}\right)\left(t_{replyB}-t_{replyA}\right)$$

Again, Equation (17) reduces to (18), if there is no delay in message delivery.

### 4.3. Extended AltDS-TWR Method

By applying Equation (6) into the proposed error model from Equations (14a)–(14d), and by assuming $T_{tof}<<t_{replyA}\;\left(\mathrm{or}\right)\;t_{replyB}$ (Section 5.1), the TOF error between the estimated and the true value for AltDS-TWR becomes:

(19) $${\hat{T}}_{tof}-T_{tof}\approx \frac{C_{1}t_{replyA}t_{replyB}}{C_{2}t_{replyA}+C_{3}t_{replyB}}$$

where $C_{1}=\xi_{BAB}\left(1+e_{A}\right)+\xi_{ABA}\left(1+e_{B}\right)+\xi_{BAB}\xi_{ABA}$, $C_{2}=2+e_{A}+e_{B}+\xi_{BAB}$ and $C_{3}=2+e_{A}+e_{B}+\xi_{ABA}$. The formula derivation is publicly available in Reference [26].

For the sake of comparison, the TOF error for the AltDS-TWR method [4] using the conventional model from Equations (9a)–(9d) is:

(20) $${\hat{T}}_{tof}-T_{tof}=e_{A}\;\cdot \;T_{tof}\;\left(\mathrm{or}\right)\;{\hat{T}}_{tof}-T_{tof}=e_{B}\;\cdot \;T_{tof}$$

Again, Equation (19) equals zero if it is assumed that $\xi_{BAB}=0$ and $\xi_{ABA}=0$. This explains that the actual TOF error is associated only with $T_{tof}$ as in Equation (20). This is because it is assumed that $T_{tof}$ is negligible ($T_{tof}<<t_{replyA}\;\left(\mathrm{or}\right)\;T_{tof}<<t_{replyB}$) when Equation (19) is formulated [26].

### 4.4. Extended ADS-TWR Method

By substituting Equations (14a), (14c) and (14d) in Equation (8), and by replacing the $t_{roundA}$ and $t_{roundB}$ with Equations (7a) and (7b), the TOF error for the ADS-TWR method can be formulated as:

(21) $${\hat{T}}_{tof}-T_{tof}=\frac{1}{2}T_{tof}\left(e_{A}+e_{B}+\xi_{ABA}+\xi_{BAB}\right)+\frac{1}{4}\left(e_{A}-e_{B}\right)t_{replyB}+\frac{1}{4}\left(\xi_{ABA}-\xi_{BAB}\right)t_{replyB}$$

For comparison, the TOF error for the ADS-TWR method using the conventional model from Equations (9a), (9c) and (9d) is:

(22) $${\hat{T}}_{tof}-T_{tof}=\frac{1}{2}T_{tof}\left(e_{A}+e_{B}\right)+\frac{1}{4}\left(e_{A}-e_{B}\right)t_{replyB}$$

## 5. Analytical Comparison of TWR Methods

In this section, we compare the analytical results of TOF error among different TWR methods. To do this, we classify three types of assumptions as defined in Section 5.1.

### 5.1. Error-Model Classification in Three Types

In order to uniformly compare the four evaluated TWR methods, we establish three assumptions (Table 1). In each of the three assumptions, it is assumed that $T_{tof}$ is negligible compared to reply time ($t_{replyA}\;\mathrm{and}\;t_{replyB}$), i.e., $T_{tof}<<t_{reply},\;t_{replyA},\;t_{replyB}$. Detailed comparison and discussion upon these three assumptions are addressed in Section 5.2, Section 5.3 and Section 5.4. The three types of assumptions (Table 1) are:

Type I Assumption: This is an ideal case. Assume $T_{tof}<<t_{reply}$, $e_{A}=e_{B}=e=0$, and $t_{replyA}=t_{replyB}=t_{reply}$. In this assumption, not only are there no clock-drift errors between the two evaluated devices, but reply times are also assumed to be the same.

Type II Assumption: This is a special case. Assume $T_{tof}<<t_{reply}$ and $t_{replyA}=t_{replyB}=t_{reply}$. In this assumption, clock-drift error does exist in the evaluated two devices. However, reply times between them are assumed to be the same.

Type III Assumption: This is a typical case. Assume $T_{tof}<<t_{reply}$ and $t_{replyA}\neq t_{replyB}$. In this assumption, not only does clock-drift error exist in the evaluated two devices, but also the reply time between them is different.

### 5.2. Comparison of TWR Methods in Ideal Cases (Type I)

According to the Type I assumption, we can conclude that $\xi_{BAB}=\xi_{ABA}=\xi$. By applying this ideal assumption to Equations (15), (17), (19) and (21), the TOF error between the estimated and true value among TWR methods can be summarized as follows:

(23) $${\hat{T}}_{tof}-T_{tof}\approx \frac{1}{2}\xi t_{reply}$$

The TOF error for all methods is now approximated as given in Equation (23). The formula derivation for AltDS-TWR is publicly available in Reference [26].

### 5.3. Comparison of TWR Methods in Special Cases (Type II)

By applying a Type II assumption in Equations (15), (17), (19), and (21), the TOF error between the estimated and true value among TWR methods can be represented as follows:

The SS-TWR method becomes:

(24) $${\hat{T}}_{tof}-T_{tof}\approx \frac{1}{2}\left(e_{A}-e_{B}+\xi_{ABA}\right)t_{reply}\;$$

The SDS-TWR method turns into:

(25) $${\hat{T}}_{tof}-T_{tof}\approx \frac{1}{4}\left(\xi_{BAB}+\xi_{ABA}\right)t_{reply}\;$$

The AltDS-TWR method is:

(26) $${\hat{T}}_{tof}-T_{tof}\approx \frac{K_{A}}{K_{B}}t_{reply}$$

where, $K_{A}=\xi_{BAB}\left(1+e_{A}\right)+\xi_{ABA}\left(1+e_{B}\right)+\xi_{BAB}\xi_{ABA}$ and $K_{B}=4+2\left(e_{A}+e_{B}\right)+\xi_{BAB}+\xi_{ABA}$. The formula derivation is publicly available in Reference [26].

The ADS-TWR method becomes:

(27) $${\hat{T}}_{tof}-T_{tof}\approx \frac{1}{4}\left(e_{A}-e_{B}+\xi_{ABA}-\xi_{BAB}\right)t_{reply}\;$$

By comparing Equation (24) to (27), we can conclude that SDS-TWR (25) and AltDS-TWR (26) are superior to SS-TWR (24) and ADS-TWR (27). The reason is that, if $\xi_{BAB}=0$ and $\xi_{ABA}=0$, the TOF error for SDS-TWR (25) and AltDS-TWR (26) is approximately equal to zero.

### 5.4. Comparison of TWR Methods in Typical Cases (Type III)

By applying a Type III assumption in Equations (15), (17), (19) and (21), the TOF error among the evaluated TWR methods is as follows:

The SS-TWR method becomes:

(28) $${\hat{T}}_{tof}-T_{tof}\approx \frac{1}{2}\left(e_{A}-e_{B}+\xi_{ABA}\right)t_{replyB}$$

The SDS-TWR method turns into:

(29) $${\hat{T}}_{tof}-T_{tof}\approx \frac{1}{4}\left(e_{A}-e_{B}\right)\left(t_{replyB}-t_{replyA}\right)+\frac{1}{4}\left(\xi_{BAB}t_{replyA}+\xi_{ABA}t_{replyB}\right)$$

The AltDS-TWR method is:

(30) $${\hat{T}}_{tof}-T_{tof}\approx \frac{C_{1}t_{replyA}t_{replyB}}{C_{2}t_{replyA}+C_{3}t_{replyB}}$$

where $C_{1}=\xi_{BAB}\left(1+e_{A}\right)+\xi_{ABA}\left(1+e_{B}\right)+\xi_{BAB}\xi_{ABA}$, $C_{2}=2+e_{A}+e_{B}+\xi_{BAB}$ and $C_{3}=2+e_{A}+e_{B}+\xi_{ABA}$. The formula derivation is available in Reference [26].

The ADS-TWR method becomes:

(31) $${\hat{T}}_{tof}-T_{tof}\approx \frac{1}{4}\left(e_{A}-e_{B}+\xi_{ABA}-\xi_{BAB}\right)t_{replyB}$$

By comparing Equations (28)–(31), we can conclude that the AltDS-TWR Equation (30) method stands out to be the best choice for minimizing TOF error. This is because the TOF error is approximately equal to zero if it is assumed that there are absolutely no delay errors in the message delivery, i.e., $\xi_{BAB}=0$ and $\xi_{ABA}=0$.

## 6. Numerical Simulation Results

In this section, we present the numerical simulation results of the proposed analytical model given in Section 5. Simulations have been performed upon the parameters, which are clock-drift errors $e_{A}$ and $e_{B}$, the reply time of responder device ($t_{replyA}$ and $t_{replyB}$), and the relative delay error in the round-trip time of a signal ($\xi_{BAB}$ and $\xi_{ABA}$), introduced in Section 3.1. The numerical sample values used for the simulations are shown in Table 2. The relative delay error in round-trip time for both transceivers is assumed to be the same, i.e., $\xi =\xi_{BAB}=\xi_{ABA}$, in the presented simulation results. Moreover, the same random seed value is used for $e_{A}$ and $e_{B}$ throughout the simulations.

### 6.1. Simulation Results for Ideal Cases (Type I)

**Figure 3:**
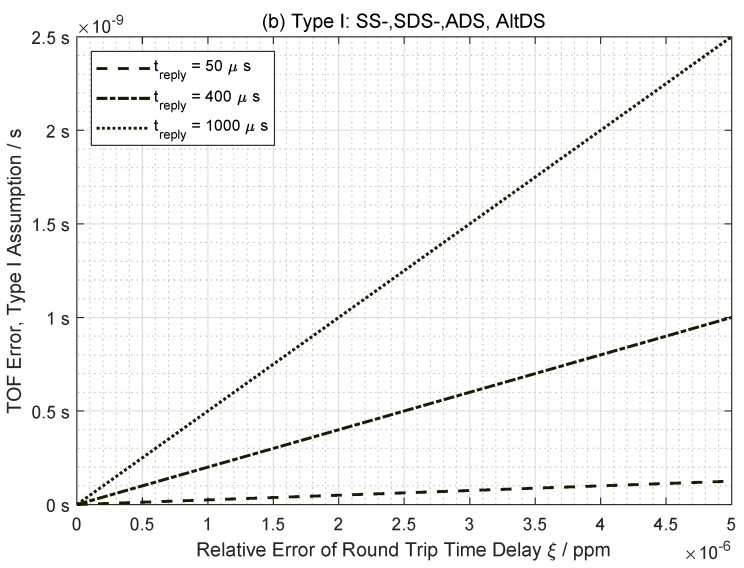
TOF error comparison using a Type I assumption (ideal case) as in Equation (23) (©2018 IEEE. Reprinted, with permission).

The ideal condition is the simplest and also the reference case because it defines how the system is expected to behave. From Figure 3, it is observed that TOF error in an ideal case increases monotonically as both round-trip time delay (ξ) and reply time ($t_{reply}$) are increased. Moreover, all TWR methods perform equally well in ideal conditions.

### 6.2. Simulation Results for Special Cases (Type II)

**Figure 4:**
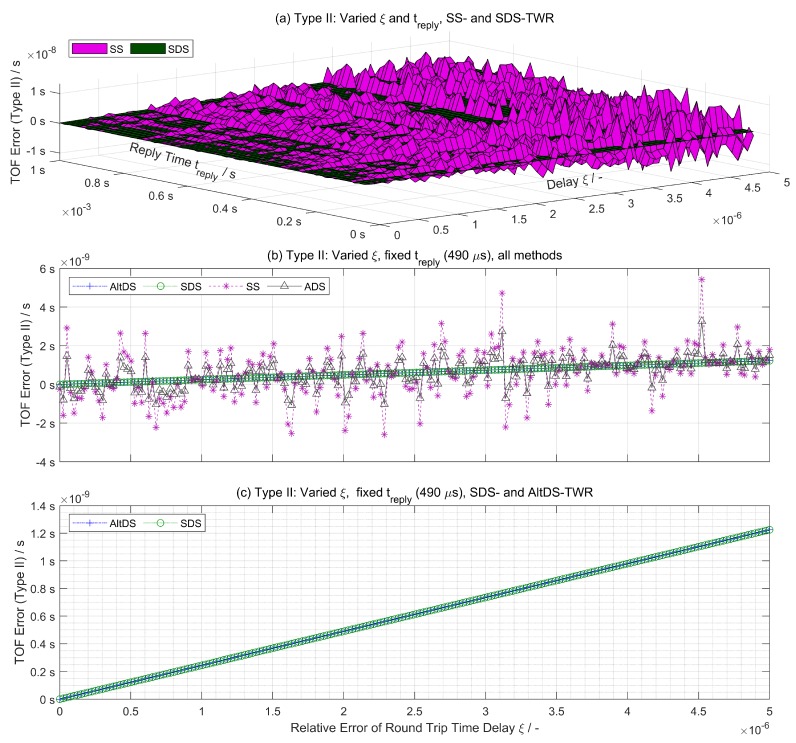
TOF error comparison using a Type II assumption (special case) in accordance with Equations (24)–(27). (**a**) TOF error for SS-TWR and SDS-TWR on 65 sample points (see Table 2); (**b**) TOF error vs. delay (ξ); and (**c**) TOF error specifically for SDS-TWR and AltDS-TWR.

A comparison between the TWR methods for special cases (Type II) is illustrated in Figure 4 relative to round-trip time delay (ξ) and reply time ($t_{reply}$). Fxed reply time $t_{reply}=490\;\mu$s is set in the simulation to match the hardware setup in the experimental evaluation (Section 7). Interestingly, it is evident that the AltDS-TWR method retains the exact same performance as the SDS-TWR method (Figure 4c).

In this special case, both the AltDS-TWR and SDS-TWR method provide numerically stable outputs for TOF error estimation (Figure 4c). In essence, TOF error in all evaluated methods is perpetually increased due to clock drifts as reply time ($t_{reply}$) and round-trip time delay (ξ) are increased (Figure 4).

According to the value of parameters used in the simulation (Table 2), the TOF error for both the SDS-TWR and AltDS-TWR method is less than 1 ns if ξ< 3 ppm and $t_{reply}<$ 650 μs. This corresponds to approximately less than 30 cm error in physical-distance measurement. Under the assumption that the round-trip time delay for both transceivers is symmetric, if ξ can be decreased to 2 ppm, then $t_{reply}$ can be relaxed up to 1 ms without the loss of the above-mentioned accuracy (30 cm). The same principle applies the other way around, too, i.e, decreasing $t_{reply}$ relaxes the increase of ξ.

### 6.3. Simulation Results for Typical Cases (Type III)

**Figure 5:**
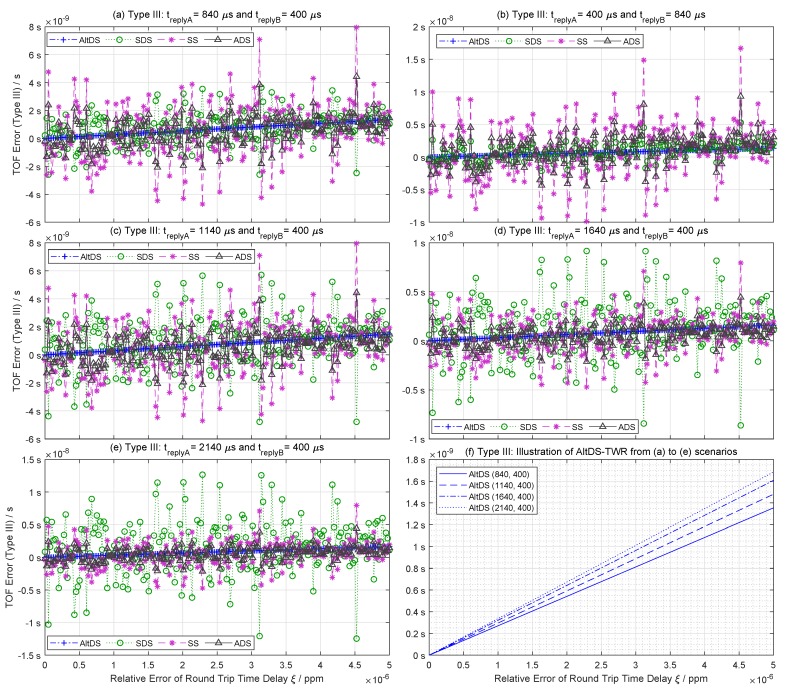
TOF error comparison between TWR methods using Type III assumption (typical case) as in Equations (28)–(31). (**a**) TOF error when $t_{replyA}>t_{replyB}$, (**b**) TOF error when $t_{replyA}<t_{replyB}$, (**c**) TOF error when $t_{replyA}=1140\;\mu s$ and $t_{replyB}=400\;\mu s$, (**d**) TOF error when $t_{replyA}=1640\;\mu s$ and $t_{replyB}=400\;\mu s$, (**e**) TOF error when $t_{replyA}=2140\;\mu s$ and $t_{replyB}=400\;\mu s$, and (**f**) TOF error specifically for AltDS-TWR method at different reply times.

The simulation results for a typical condition (Type III) between the four evaluated TWR methods are provided in Figure 5. Figure 5a compares the performance of the TWR methods when the reply time in Device A ($t_{replyA}=840\;\mu s$) is greater than the reply time in Device B ($t_{replyB}=400\;\mu s$). In contrast, Figure 5b compares the performance of the TWR methods when the two reply times are in the opposite order ($t_{replyA}<t_{replyB}$) by switching the value of the mentioned reply times. Figure 5c–e illustrates the variation of TOF error in SDS-TWR upon different reply times. The reply-time values in the simulation (Figure 5) were chosen to match with the hardware setup in the experimental evaluation (Section 7).

It is evident that the SDS-TWR method suffers severe clock-drift error effects in a typical condition (Type III) when the reply time is asymmetric (Figure 5a–e). However, the AltDS-TWR method still holds a numerically stable result in each evaluation (Figure 5f).

Note that the ADS-TWR and SS-TWR methods rely solely on one-sided reply time ($t_{replyB}$). Therefore, the duration of $t_{replyB}$ is crucial for their performance. On the one hand, when $t_{replyB}<t_{replyA}$, the ADS-TWR method yields a lower TOF error than the SDS-TWR method, while SS-TWR has a fairly comparable result (Figure 5a). On the other hand, when $t_{replyB}>t_{replyA}$, the performance of the SS-TWR and ADS-TWR methods degrades, while the performance of the SDS-TWR method is unchanged. In this scenario, the TOF error in the SDS-TWR method is lower than both the SS-TWR and ADS-TWR method (Figure 5b). The severity of the TOF error in SDS-TWR increases as the magnitudes of difference between the two reply times increases (Figure 5a–e).

## 7. Experimental Evaluation Results

The experimental evaluations of the three TWR methods, namely, SS-TWR, SDS-TWR, and AltDS-TWR, are conducted in this section. Note that the ADS-TWR method is not included in the experimental evaluation because the hardware used in the experiment doesn’t support the necessary mechanism for ADS-TWR (Figure 2) at the time of our evaluation, which is the instant reply time in Device A ($t_{replyA}=0$) or an autoacknowledgment mechanism in one of the two devices.

**Figure 6:**
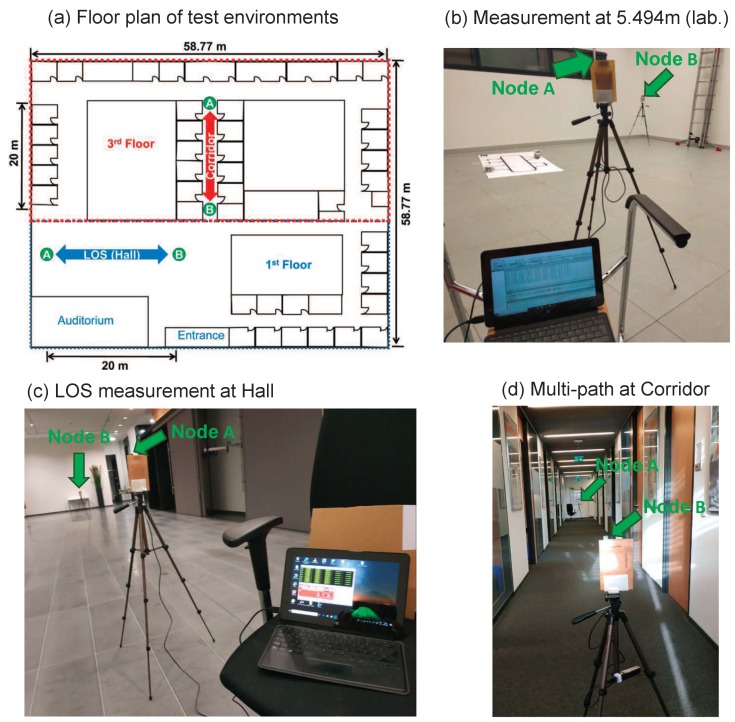
Test environments of the experimental evaluations: (**a**) overview of office floor plan for the LOS experiment in hall (blue arrow) and the multipath experiment in a corridor (red arrow), (**b**) fixed-distance experiment in the laboratory, (**c**) LOS experiment in a hall (office environment), and (**d**) multipath experiment in a corridor (office environment).

This section is categorized into three parts. The first part is the experiment setup, where the hardware and its corresponding configurations used in the evaluation are introduced. In the second part, the experiment results for fixed reply times at different locations (LOS at a hall, a multipath scenario at the corridor in an office building, and close LOS less then 2 m) are expressed. The goal is to clarify the errors caused by the delays (PTD and PATD) as mentioned in Section 3.1. In the third part, comparative analysis between three TWRs is conducted at a fixed location (distance) in the laboratory with varying reply times. The goal is to point out the pitfalls of the SDS-TWR method in a typical case, and to prove that AltDS-TWR holds stable results in each reply-time variation. The test environments where the experimental evaluations presented in this section were conducted are illustrated in Figure 6.

### 7.1. Setup and Data-Collection Process for Experimental Evaluations

For experimental evaluations, we used a DWM1000 module [27] from Decawave as the UWB hardware, and an STM32 development board (NUCLEO-L476RG) from STMicroelectronics as the main microcontroller (MCU). Moreover, the built-in high-speed internal (HSI) clock source (16 MHz) from the MCU was applied to all of the evaluation results presented in this article. No external oscillators were connected to the MCU. The HSI has an accuracy of ± 1 using the factory-trimmed RC oscillator according to the datasheet [28].

Aggregated antenna delay calibration was conducted before measurement according to the procedure and algorithm provided by the manufacturer [29,30]. This aggregated antenna delay corresponds to transmission and receiving time delays (TTD and RTD) of the evaluated hardware described in Section 3.1. Therefore, the remaining error that influences the accuracy of TOF error estimation in our measurement would be PTD and PATD. The results presented in Section 7 are the errors and their corresponding parameters in distance (not in TOF). This is because all of the references used in the experiment are measured in distance, which means that TOF value is already calculated as a distance by multiplying with the speed of light (299,702,547 ms^−1^ in air).

During measurement, one of the transceivers (Device A in Figure 1) is connected to a computer for logging the data received from the MCU via serial USART port. Two-way ranging software, provided by Decawave for production testing of their evaluation kit (EVK1000), which is available online (https://www.decawave.com/software/) on Decawave’s website, was executed on the two transceivers. The software was modified so that the four periods of time ($t_{roundA},\;t_{roundB},\;t_{replyA},\;\mathrm{and}\;t_{replyB}$) were individually logged and saved into a file at each measurement. The above-mentioned time periods from the log file were afterward processed with the TWR formulas provided in Section 2 using Matlab. This ensured that the same raw data (time periods) were used for the three TWRs in the evaluation. For instance, a subset of the four collected time periods, i.e., $t_{roundA}$ and $t_{replyB}$, was used to study SS-TWR.

All of the reference distances in the evaluation were measured with a laser distance meter, CEM iLDM-150 model (http://www.cem-instruments.in/product.php?pname=iLDM-150), which has an accuracy of ±1.5 mm according to the manufacturer. The hardware configuration of the used UWB module in the experimental evaluations is described in Table 3. Antenna height was 1.06 m in all the experiments reported in this paper. This ensured that the effect of Fresnel zones did not perturb measurement results.

**Figure 7:**
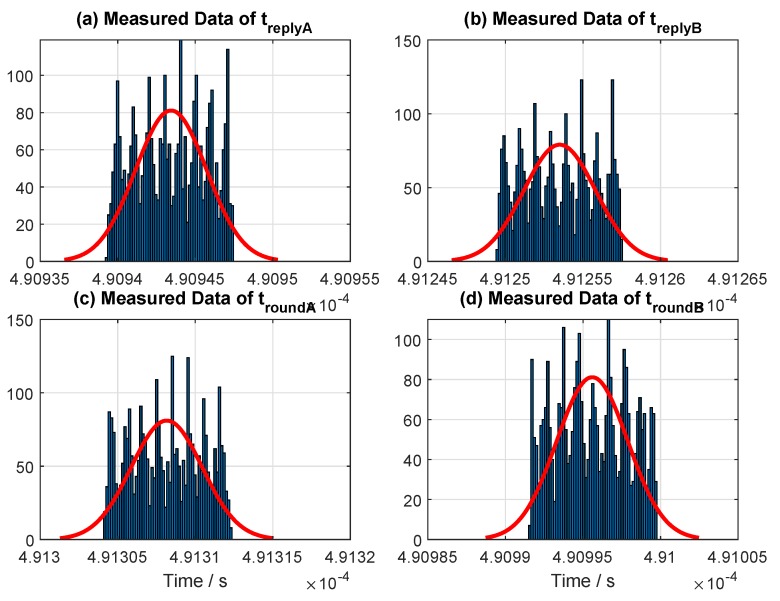
Measured data for fixed reply times used in the experiments for the special case (Type II).

For a symmetric condition in special cases (Type II), the hardware for the two transceivers was tuned until the two reply times were approximately equal (symmetric). The histogram of the sample data for symmetric replied time (special case, or Type II) collected from one of our measurements is shown in Figure 7. The figure shows the measured time periods for a single trial conducted roughly around 5 min with an updated rate of 10 Hz. The mean values of the reply times are $t_{replyA}=490.94\;\mu s$ and $t_{replyB}=491.25\;\mu s$ (Figure 7 and Table 4). This setting and reply time were used throughout all of the evaluation results presented in this paper for a symmetry case (Type II).

**Figure 8:**
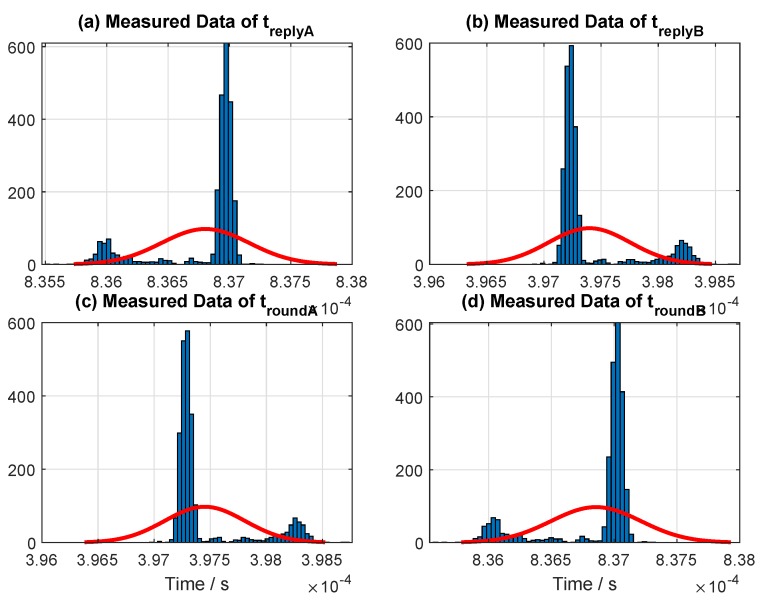
Measured data for fixed reply times used in the experiments for a typical case (Type III).

For an asymmetric condition in a typical case (Type III), the histogram of the sample data collected from one of our measurement is illustrated in Figure 8. Again, the figure illustrates the measured time periods for a single trial. The mean values of the reply times are $t_{replyA}=836.8\;\mu s$, and $t_{replyB}=397.4\;\mu s$ (Figure 8 and Table 4). Note that this is the default setup (out of the box) achieved from the software provided by Decawave. This setting and reply times are used for the measurement conducted in LOS (hall), multipath (Corridor), and close LOS. However, reply time was varied on one device at each evaluation conducted in Section 7.3 to compare the performance difference between the SDS- and AltDS-TWR methods.

Table 4 represents the sample data of reply times for Types II and III, which were randomly drawn from the measurement conducted in the three categories, at LOS, close LOS, and multipath scenarios. It was confirmed that the magnitude of difference (similarity) between the two reply times ($t_{replyA}\;\mathrm{and}\;t_{replyB}$), which is annotated as root mean square error (RMSE) in Table 4, for the symmetry case (Type II) in all of our measurements was always less than 0.35 μs in average.

### 7.2. Comparative Analysis of Distance Errors in Fixed Reply Times at Different Scenarios

In this subsection, experimental evaluations of three scenarios, that is, close LOS, LOS (Hall), and multipath (Corridor), were conducted to validate the error influenced by the PTD and PATD. The effect of PTD can be seen in the multipath scenario, where measurement was conducted in the corridor of an office building (Section 7.2.1), and in the Non-LOS (NLOS) scenario (Section 7.3.2). In the same way, the effect of PATD can be seen in the close LOS scenario, where measurement was conducted within less than 2 m (Section 7.2.2). The complete detailed report of the three scenarios is presented in Section 7.2.3 (see Table 5 for the special case (Type II) and Table 6 for the typical case (Type III)).

#### 7.2.1. Distance Error Comparison for Types II and III at LOS and Multipath Scenarios

To evaluate the distance error caused by the effect of a multipath signal in TWR, measurement was conducted at different ranges for both an LOS scenario (Figure 6c) where measurement was done in a big hall, and a multipath scenario (Figure 6d) where measurement was conducted in the narrow corridor of an office environment. Note that the UWB signal natively overcomes the multipath effects compared to other narrow-band signals. However, signal disturbance because of multipath effects in UWB is still noticeable in distance error estimation, as is shown in the following paragraphs.

**Figure 9:**
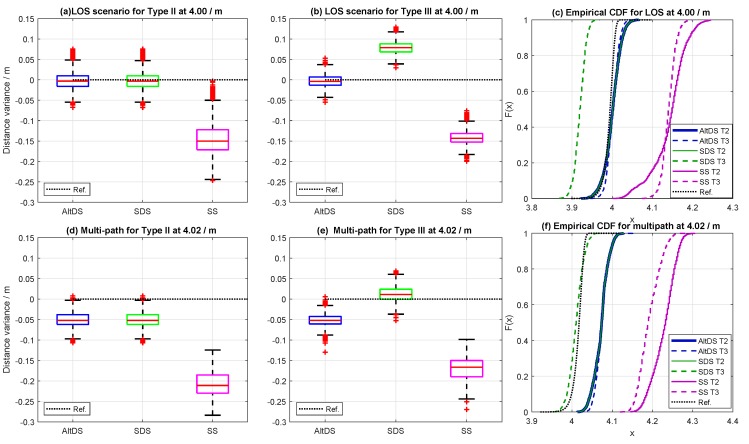
Comparison of Type II and Type III for LOS and multipath scenarios at a 4 m true reference. (**a**–**c**) measured data from the LOS (Hall), and (**d**–**f**) measured data from the multipath (Corridor) scenario.

Figure 9 depicts the measurement results for the exact same distance (4 m) for two separate scenarios (LOS at hall and multipath at corridor). The first row, Figure 9a–c, illustrates the results achieved from the LOS condition, and the second row, Figure 9d–f, illustrates the results achieved from the multipath scenario.

Furthermore, the measured results for both Type II and III are compared side by side in Figure 9 to clearly see the differentiation between the two cases. It can be seen in the experiment result that SDS and AltDS have approximately the same performance level in the special case (Type II) as already stated in the simulation results (Section 6, see Figure 9a,d). However, a significant variation between SDS and AltDS can be observed in the typical case (Type III) as expected from the simulation results (Section 6, see Figure 9b,e).

Regarding distance error in the TWR approach, it was observed that both SDS and AltDS outperformed SS-TWR with significant distinction in all cases (Figure 9). Particularly for the LOS scenario (first row in Figure 9), the measured distances of both SDS and AltDS were very close to the reference value in Type II (Figure 9a). However, AltDS had the smallest error between the three in the typical case (Figure 9b). In the multipath scenario (second row in Figure 9), the distance error caused by SDS and AltDS was still small compared to that of SS-TWR in the special case (Figure 9d), even though their errors were slightly higher compared with the LOS case (Figure 9a,d). In the typical case (Type III), the figure suggests that SDS had the smallest error beetween the three methods (Figure 9e). This condition is further analyzed for clarity in Section 7.3.

In general, the multipath effect caused a big data shift in all of the measurements for all three of the evaluated methods. This shift can clearly be seen by comparing the empirical cumulative distribution function (eCDF) for the LOS scenario, presented in Figure 9c, and for the multipath scenario, presented in Figure 9f. This corresponds to the contribution of the delay caused by the multipath signal in distance or TOF error estimation as stated in Section 3.1. This delay could be the PTD because of the reflection of the signal as well as the PATD in the case of multiple signals arriving within the chip period of the first path signal, as described in Section 3.1.

**Figure 10:**
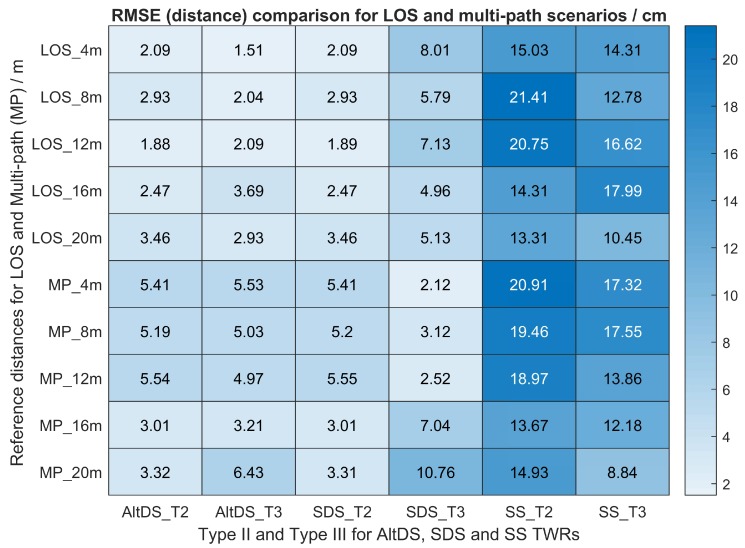
Measured distance error comparison of Types II and III for three TWRs evaluated in different locations in LOS and multipath scenarios.

Figure 9 gives inside knowledge for visualizing the experimental data for the two scenarios (LOS and multipath), specifically measured at true reference 4 m. The complete dataset for Types II and III at different ranges in two scenarios (LOS and multipath) is provided in Figure 10. The data in Figure 10 represent the RMSE, which is the square root of the mean error between the measurement and the true reference of the special and typical cases (Types II and III) for the three evaluated TWR (AltDS, SDS, and SS) at different locations in two scenarios (LOS and multipath).

In general, it was observed that the distance error for AltDS was less than 6.43 cm in all of the measurements at both the special case (Type II) and the typical case (Type III) (see the first two columns in Figure 10). Moreover, the measured distance error for all locations in AltDS and SDS was approximately equal in the special case (Type II) (see the first and third columns in Figure 10). Obviously, the largest distance errors in the measurement occurred in SS-TWR (Figure 10).

In the multipath scenario at Type III (esp. 4, 8, and 12 m), the figure suggests that the distance error in SDS provides the smallest among the three evaluated methods (the fourth column in Figure 10). This happens because of the chosen fixed reply time ($t_{replyA}=836.8\;\mu s$ and $t_{replyB}=397.4\;\mu s$) for a typical case in this particular multipath condition. The issue is further clarified by varying the reply time of one device using different values in the measurement (see Section 7.3).

#### 7.2.2. Distance Error Comparison for Types II and III at a Close LOS Scenario

To evaluate the PATD effect, measurement for close LOS (measured distances range from 0.25 up to 2 m) was conducted at one of the CITEC laboratories, Bielefeld University (Figure 6b). PATD occurrence is significant in close LOS, especially when a coherent receiver architecture is used in the hardware [22]. The reason is that a sequence of preamble code is necessary to synchronize in the physical layer before data communication between transceivers can be started using the property of perfect periodic autocorrelation [3,22]. Moreover, most of the commercially available UWB hardware modules, including DWM1000, used in this evaluation are based on a coherent receiver structure. In this experiment evaluation, preamble sequence code index no. 3 was used, which has the code sequence pattern of “−+0++000−+−++00++0+00−0000−0+0−” according to References [3,22] (p. 203). This sequence is regarded as a short one in UWB configurations. It is expected that, the longer the code sequence is, the more likely to have severe error in close LOS measurement. The reason is that the base symbol rate for the synchronization header is proportional to the preamble symbol transmission rate [3] (pp. 200–207). This means that the longer the preamble length is, the longer it takes to detect the start of frame delimiter (SFD) during accumulation time.

**Figure 11:**
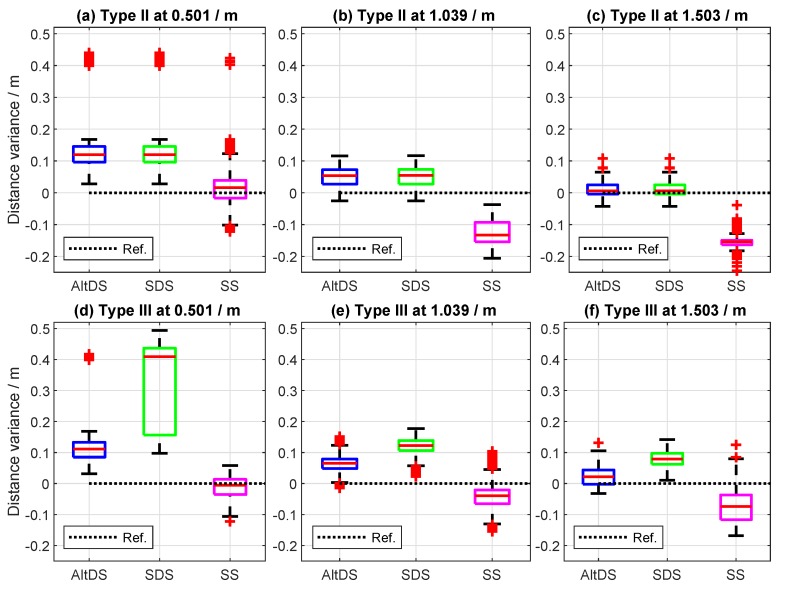
Results comparison of Types II and III for close LOS at true reference 0.25, 1.00, and 1.50 m. (**a**–**c**) measurement results for Type II (special case), and (**d**–**f**) corresponding results for Type III (typical case).

**Figure 12:**
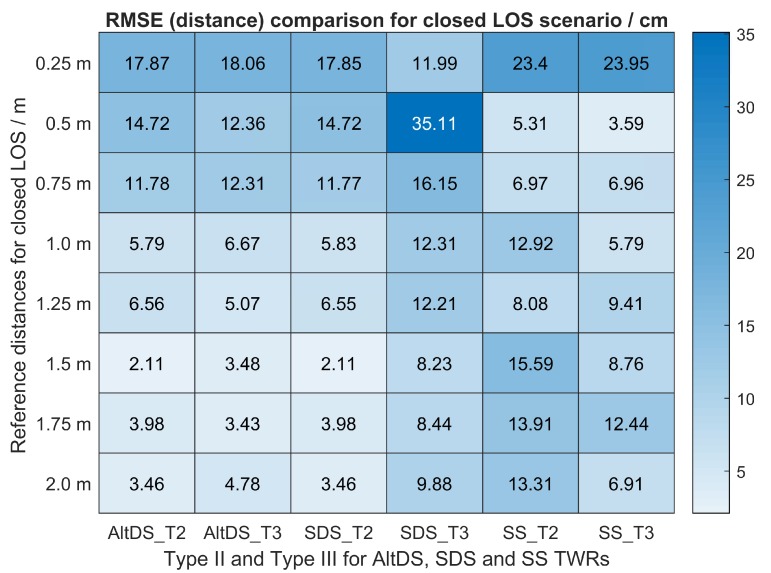
Measured distance error (RMSE) comparison of Types II and III for three TWRs in a close LOS scenario.

Regarding this, data visualization using boxplots for Types II and III at a true reference of 0.5, 1.0, and 1.5 m is presented in Figure 11. The RMSE regarding measured distance error for the three evaluated TWRs in Types II and III, conducted at close LOS, is provided in Figure 12.

In general, a significantly high rate of outliers (symbolized with red plus signs) is presented in the data of measurement results less than 0.75 m (see the sample data measured at 0.5 m (Figure 11a,d). It can also be seen that the measured SDS data relatively skew at a 0.5 m reference (Figure 11d). The contribution of outliers in the data decreases as the true-reference distances between the two transceivers increase. Specifically, the RMSE for all TWRs in both cases was roughly greater than 11 cm at reference distances less than 0.75 m (first three rows in Figure 12). Moreover, it can be stated (for this particular UWB setup) that the effect of the PATD on the LOS condition decreases, starting from 1.5 m of the true reference (last three rows in Figure 12). In this case, the measured RMSE for AltDS (both Type II and Type III) and SDS (Type II) is always less than 5 cm. The transition phase (RMSE between 5 and 10 cm) can be spotted in the measured results at the true reference of somewhere between 0.75 and 1.5 m.

#### 7.2.3. Detailed Summary of Experiment Results for Fixed Reply Time

The detailed summary of the measurement results reported in Section 7.2.1 and Section 7.2.2 is expressed in Table 5 (Type II) and Table 6 (Type III). The smallest value between the three methods is presented in bold in the tables. Two numbers less than 0.02 cm apart were considered equal. It is interesting to observe that the data spread (the difference between maximum and minimum values in the recorded data) in the symmetry case (Type II) was less than 10 cm for both SDS and AltDS when measured in the LOS scenario (rows of “LOS” and columns of “Spread of data” in Table 5). That is the best-case scenario spotted in the measurement. In fact, this condition matches the reported precision range of the manufacturer, i.e., 10 cm. In the worst-case scenario, the data spread reaches up to 41.23 cm in both AltDS and SDS TWRs, while it reaches up to 54.54 cm in SS-TWR. As a whole, the spread of data generally increased in the typical case (Type III) (see Table 5 and Table 6). Moreover, the measured data were also spread wider, in general, in both the multipath and the close LOS scenario (Table 5 and Table 6).

### 7.3. Comparative Analysis of Distance Error in Variable Reply Times at a Fixed Distance

This section mainly focuses on comparative analysis between AltDS and SDS TWR (Section 7.3.1). The goal is to demonstrate SDS pitfalls with an experimental evaluation. At the same time, it also aims to prove, with experiment results, the efficiency of the AltDS method in a typical case when reply times are varied. Additionally, the effects of NLOS on distance error are also reported in Section 7.3.2 to assure the influence of PTD on the measurement.

#### 7.3.1. Distance Error Analysis between SDS and AltDS at Fixed Reference

**Figure 13:**
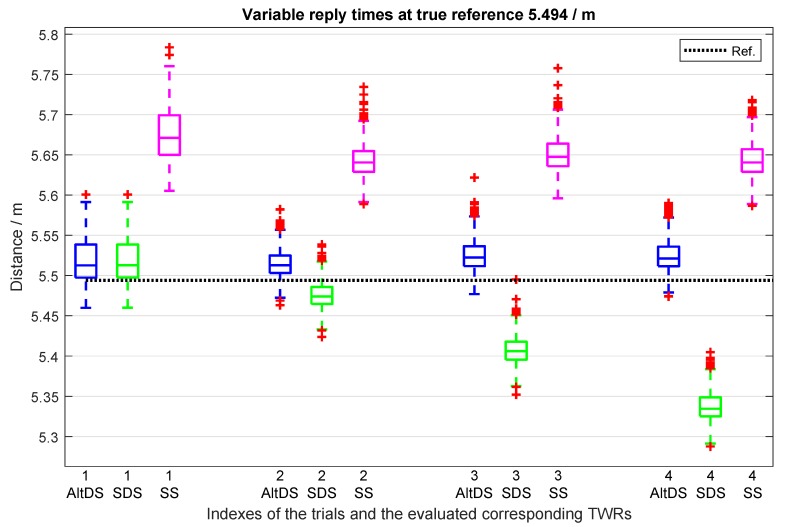
Variable reply times at fixed distance measurement.

A total of ten trials for variable reply times with the magnitude of difference between the two reply times ($t_{replyA}\;$and$\;t_{replyB}$) starting from 0.0003 (symmetry case) up to 4.2 ms are provided in (Table 7). The first four trials from Table 7 are illustrated in Figure 13 to visualize the data for better analysis. Note that only one of the two reply times, specifically, $t_{replyA}$, is varied in the experiment by manipulating the value of delays on the ranging software (see Columns 6 and 7 from Table 7, denoted as “Reply Time (ms)”).

The symmetry case is again evaluated in this scenario as provided in Trial 1 to demonstrate comparative analysis between the special case (Type II) and typical case (Type III). In the symmetry case, both AltDS and SDS had identical performance level and the RMSE in distance estimation was also the same in this particular experiment, with an exact value of 3.44 cm (Figure 13 and the first-row in Table 7). A similar result was already reported in Section 7.2. However, distance error using SS-TWR is significantly higher than both of AltDS and SDS.

Figure 13 also clearly demonstrates the pitfall of SDS-TWR, frequently mentioned throughout this article. As the name symmetric is applied in the method itself, a very small error occurs in SDS when the two reply times are exactly the same (Trial 1 in Figure 13). Moreover, we witnessed that SDS is comparable to AltDS in the symmetry case. Note that it rarely happens to have a symmetric reply time in a real-world situation. In this experiment, the two reply times ($t_{replyA}$ and $t_{replyB}$) were tuned to the software until they approximately had the same value (see the tuned value of their magnitude of difference ($t_{replyA}-t_{replyB}$)) in Table 7 (second column), which had 0.0003 ms in symmetry case (first row). When symmetry in the reply time is broken down, SDS encounters significant distance error. The severity in error monotonously increases as the difference between the reply times ($t_{replyA}-t_{replyB}$) increases (Figure 13 and Table 7). Distance error even reaches 50.75 cm when the the magnitude of difference between the two reply times ($t_{replyA}-t_{replyB}$) is 4.24 ms (last row in Table 7).

It is interesting to observe that variation in the reply time ($t_{repyA}$ and $t_{replyB}$) does not affect distance error in the AltDS-TWR method. The consistency in distance error using AltDS-TWR across several trials is illustrated in Figure 13, and the exact numerical values are provided in Table 7. The distance error in RMSE for AltDS-TWR ranges in a consistent manner throughout the measurement. The exact value of RMSE varies from 2.74 to 3.72 cm, which is very small, when the magnitude of difference between reply times ($t_{replyA}-t_{replyB}$) varied between 0.0003 and 4.24 ms (Table 7). This proves the consistency of distance estimation in AltDS-TWR, which is very useful for multiple application areas. For instance, the presented delay could be thought of as a nondeterministic sensor-data reading and processing time in body area network application, where both positioning and sensor data are necessary to be loaded onto the payload of the UWB MAC layer protocol.

Regarding the SS-TWR, it can be examined that distance error is consistent across the evaluated trials (Figure 13). The reason is that SS-TWR is based only on one reply time, namely, $t_{replyB}$ (2). In the experiment, only one reply time ($t_{replyA}$) was varied, and the other reply time ($t_{replyB}$) was approximately constant throughout the evaluation (see the seventh column in Table 7).

#### 7.3.2. Distance Error Comparison for Type II and Type III in NLOS Scenario

**Figure 14:**
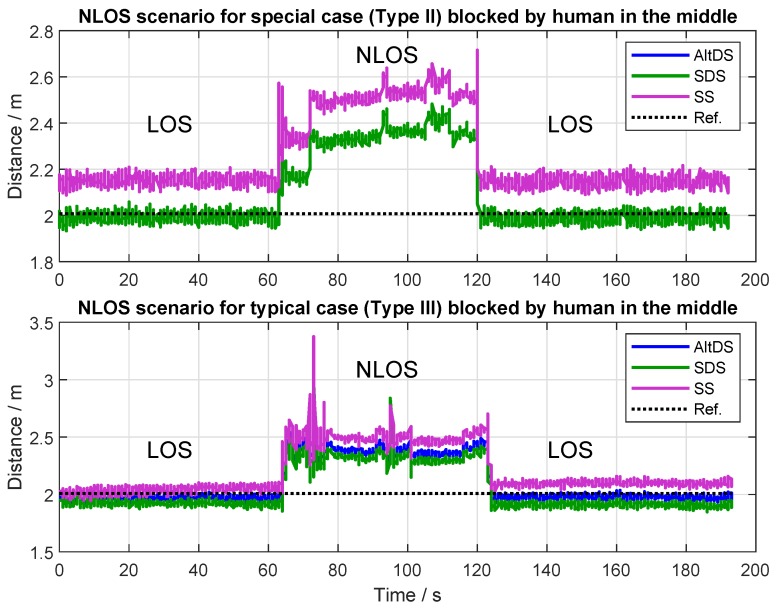
NLOS scenarios for Types II and III at 2 m when a human subject is blocking.

In order to have a complete measurement report, the NLOS scenario was also evaluated in the experiment. In this scenario, the evaluation was recorded for roughly three minutes in total for both Type II and Type III cases at a reference distance of 2 m. As it is shown in Figure 14, pure LOS data were recorded in the first minute. Then, a human subject blocked the communication between the two transceivers by standing in the middle at a distance of 1 m. After that, the blocking was removed and the pure LOS is recorded again for the remaining one minute. The mean reply times used in this evaluation were $t_{replyA}=490.94\;\mu s$ and $t_{replyB}=491.25\;\mu s$ for the symmetric case (Type II), and $t_{replyA}=836.80\;\mu s$ and $t_{replyB}=397.40\;\mu s$ for the asymmetric case (Type III), respectively.

As expected, the NLOS condition caused huge error in the measurement (roughly around 40 cm) at both of the two types in all of the evaluated TWRs (Figure 14). This shows that signal-traveling time was obstructed by the reflected signals from the environment and/or the penetration of the human body in this particular NLOS case. Therefore, it is absolutely necessary that NLOS identification and mitigation techniques are mandatory for an application where NLOS is expected to exist. It can also be seen that there were some spikes in Figure 14, which was caused by the body movement of the human subject during measurement. In the special case (Type II), both AltDS and SDS had the same measurement results, as can be seen in the upper graphic of Figure 14, in which AltDS results (blue color) were merged into the SDS (green color). In the typical case (Type III), diversity among the three TWR methods is visible, as shown in the bottom graphic of Figure 14.

## 8. Discussion

In the last decade, SDS-TWR was the most highlighted TWR technique in the literature. The reason is that it provides an impressive output of TOF error against clock drifts when reply times ($t_{replyA}$ and $t_{replyB}$) are symmetric. SDS-TWR was so popular that it even became a kind of standard. This means that, whenever a new TWR technique emerges, its performance is benchmarked with SDS-TWR as in References [4,7,8,9,10,11]. However, SDS-TWR only provides robust TOF outputs when reply times are symmetric. When the symmetry in reply time is unachievable, it encounters severe error effects (Figure 5 and Figure 13, Table 7). In fact, this is a major drawback and the pitfall of the SDS-TWR method. The constraint on strict symmetry in SDS-TWR has also been addressed in References [7,31].

In contrast, we found that AltDS-TWR method was robust against clock drift in any tested condition (Figure 4, Figure 5 and Figure 13 and Table 7). In general, minimal reply times ($t_{replyA}$ and $t_{replyB}$) and relative delay errors of round-trip time ($\xi_{ABA}$ and $\xi_{BAB}$) are desirable at any condition in every evaluated TWR method. The reason is that, the shorter the mentioned time in the system is, the better the ranging accuracy (Figure 4 and Figure 5). Note that the default (out of the box) setup achieved from Decawave’s software was used in the typical-case (Type III) experiments in LOS, close LOS, and multipath scenarios, except for the variable reply time conducted in Section 7.3.1.

Nonetheless, we proved that the corrupting effect of the delay in reply time does not affect distance-estimation accuracy in AltDS-TWR (Section 7.3.1). This point is important because the current state of the art in UWB-based localization systems only focuses on the accuracy of the positioning algorithm. The available resource space of the data payload section in MAC layer [3] (p. 57), [22] (p. 220) are not used in most of the system-level applications presented in the literature. To mention a few as examples, the system implementation of UWB in References [32,33,34,35] mainly focus on the accuracy of the positioning algorithm by assuming that no payload is included in data communication except for necessary timestamps to be used in the positioning algorithm. Therefore, if the sensor data should also be transmitted, secondary wireless technology is used in such a system.

AltDS-TWR uncovers the ability to provide both positioning and sensor data on the payload of a single UWB system without loss of accuracy in the positioning algorithm. This further ensures that AltDS can be used in sensor systems with nondeterministic processing time. For instance, variation of reply time, provided in Table 7 in Section 7.3.1, can be thought of as a representation of sensor data processing time for wireless communication systems. As an example application, AltDS-TWR can be used in a UWB-based body-sensor network in sports analysis, where position and sensor data from a mobile node are necessary to access in the central server (coach) and analyze the performance of the athlete. Note that it could take unpredictable processing time for reading the data from the sensor depending on the hardware in such a scenario.

Moreover, AltDS-TWR is preferred in real-world scenarios because it is robust against variation of reply time and clock drifts (Section 6 and Section 7). Therefore, we conclude that the performance of the AltDS-TWR method is the most reliable between the four evaluated TWR methods, under the evaluation made in our numerical and experimental development at different scenarios.

It should also be noted that the relative error presented in the simulation results (Section 6) includes all delays (PTD, PATD, TTD, and RTD) mentioned in Section 3.1. However, the relative errors regarding TTD and RTD were excluded in the experiment results (Section 7). In practice, a certain approximately constant delay error (with regard to a complete hardware setup) can be identified by evaluating two transceivers under the known ground-truth reference distance in a pure LOS scenario. The procedure of eliminating this constant, which is subtracting the mentioned constant from the measured (estimated) TOF value to match with the ground-truth reference distance, is typically called an aggregated antenna delay calibration in the world of UWB hardware implementation [29]. This corresponds with the elimination of TTD and RTD delays in our case, which was calibrated before measurement was started, as stated in Section 7.1.

Based on the evaluation results presented in this paper, a few recommendations can be drawn as follows: Firstly, distance should not be smaller than 0.75 m. The anchor (fixed) nodes should be placed far away from the dedicated region so that the distance between anchor and mobile nodes is always greater than 0.75 m. Secondly, a fairly small distance error (less than 10 cm) from both AltDS- and SDS-TWRs can be achieved if the difference between the reply times ($|t_{replyA}-t_{replyB}|$) is less than 400 μs. Thirdly, AltDS-TWR is the only solution in the evaluated TWRs when the nondeterministic processing time (e.g. sensor-data reading) are expected in the positioning system.

## 9. Conclusions and Future Work

In this paper, we proposed a novel TEEM for TWR methods. The proposed model completely holds the characteristics of the conventional clock-drift error model in TWR methods (Section 4), as defined in Reference [3]. We found that, under ideal conditions, all TWR methods perform equally well (Section 6.1). Subsequently, we showed that AltDS-TWR is superior to SDS-TWR under conditions found in typical applications (Section 6 and Section 7). SDS-TWR produces smaller TOF error against clock drifts only when reply times ($t_{replyA}$ and $t_{replyB}$) are symmetric. In contrast, AltDS-TWR provides the same level of accuracy to SDS-TWR in the symmetric case, and the most robust solution out of any of the three evaluated conditions in the asymmetric case (Section 6 and Section 7).

Distance error estimation between two transceivers was evaluated in this article. For future work, the error caused by multiple anchors (fixed-reference) nodes and a single mobile node will be analyzed. This corresponds to error analysis in a positioning algorithm between multiple anchor nodes and a single mobile node using AltDS-TWR as a ranging method.

## Figures and Tables

**Table 1:** Three assumption types for time-of-flight (TOF) Classification Errors (©2018 IEEE. Reprinted with permission).

Types	Round-Trip Delay	Clock Drifts	Reply Time
Type I	$\xi =\xi_{BAB}=\xi_{ABA}$	$e_{A}=e_{B}=0$	$t_{replyA}=t_{replyB}$
Type II	$\xi_{BAB},\;\xi_{ABA}$	$e_{A},\;e_{B}$	$t_{replyA}=t_{replyB}$
Type III	$\xi_{BAB},\;\xi_{ABA}$	$e_{A},\;e_{B}$	$t_{replyA}\neq t_{replyB}$

**Table 2:** Sample Values used in Numerical Simulations (©2018 IEEE. Reprinted with permission).

Parameters	Symbols	Range of Value	Unit
Relative delay error in round-trip time	$\xi =\xi_{BAB}=\xi_{ABA}$	0:0.025:5	ppm
Reply times in responder device	$t_{reply}=t_{replyB}$	0:5:1000	μs
	$t_{replyA}$	0 :11:2200	μs
Clock-drift error	$e_{A}$, $e_{B}$	±20 as stated in	ppm
(pseudorandom)		802.15.4-2011 [3]	

**Table 3:** Used Ultrawide Bandwidth (UWB) configuration in the evaluations.

Properties	Values
Data rate	6.8 Mbps
Channel	2
Center frequency	3993.6 M Hz
Bandwidth	499.2 M Hz
Pulse-repetition frequency (PRF)	16MHz
Preamble code sequence index [3] (p. 203)	3
Module name	DWM1000
Manufacturer	Decawave
Reported precision [27]	10 cm

**Table 4:** Sample reply time drawn randomly from each of the three categories (LOS, close LOS, and multipath scenarios). Note: RMSE, Root Mean Square Error.

Cases	RMSE (μs)	Mean (μs)		STD (ns)		Data Spread (ns)		Sample Size
	($t_{\mathrm{replyA}}-t_{\mathrm{replyB}}$)	$t_{\mathrm{replyA}}$	$t_{\mathrm{replyB}}$	$t_{\mathrm{replyA}}$	$t_{\mathrm{replyB}}$	$t_{\mathrm{replyA}}$	$t_{\mathrm{replyB}}$	
Special case	0.31	490.94	491.25	2.29	2.32	8.17	8.00	2350
(Type II)	0.28	490.97	491.25	2.30	2.32	8.47	8.00	2450
	0.26	491.0	491.25	2.34	2.34	9.14	8.00	2000
Typical case	439.41	836.80	397.40	357.14	357.11	1754.5	1754.0	2350
(Type III)	439.58	836.90	397.33	375.07	375.07	4451.3	4451.6	2450
	439.83	837.04	397.22	1369.1	1369.1	16,474.0	16,474.0	2000

**Table 5:** Experiment evaluation results for the special case (Type II) at different scenarios.

Cases	Ref. (cm)	RMSE (cm)			Standard Deviation (cm)			Spread of Data (cm)			Sample Size
		*AltDS*	*SDS*	*SS*	*AltDS*	*SDS*	*SS*	*AltDS*	*SDS*	*SS*	
Close	25.63	17.87	**17.85**	23.40	3.41	3.41	**2.00**	30.56	30.56	**9.85**	2000
LOS	50.08	14.72	14.72	**5.30**	6.85	6.85	**5.19**	**41.23**	**41.23**	54.54	2000
	75.44	11.78	11.77	**6.95**	**2.34**	**2.34**	5.05	**13.19**	**13.19**	20.06	2000
	103.90	**5.79**	5.83	12.92	**2.79**	**2.79**	3.59	**14.19**	**14.19**	16.89	2000
	125.18	**6.56**	**6.55**	8.08	**2.80**	**2.80**	4.96	**15.95**	**15.95**	24.62	2000
	150.30	**2.11**	**2.11**	15.59	1.84	1.84	**1.59**	**15.06**	**15.06**	20.64	2000
	175.27	**3.98**	**3.98**	13.91	**3.88**	**3.88**	4.09	**16.18**	**16.18**	17.53	2000
	200.77	**3.46**	**3.46**	13.31	**2.19**	**2.19**	2.40	15.71	15.71	**14.71**	2000
LOS	399.82	**2.09**	**2.09**	15.03	**2.08**	**2.08**	4.13	**14.30**	**14.31**	24.33	2450
(Hall)	806.70	**2.93**	**2.93**	21.41	**1.33**	**1.33**	**1.34**	**8.44**	**8.44**	11.73	2450
	1206.20	**1.88**	**1.89**	20.75	**1.32**	**1.32**	1.53	**9.03**	**9.03**	10.55	2450
	1600.20	**2.47**	**2.47**	14.30	**2.15**	**2.15**	9.29	**13.59**	**13.60**	40.34	2450
	2002.00	**2.00**	**2.00**	14.56	**1.97**	**1.97**	6.16	**16.17**	**16.18**	34.94	2450
Multipath	402.12	**5.41**	**5.41**	20.91	**1.87**	**1.87**	2.96	**11.43**	**11.43**	15.95	2350
(Corridor)	802.05	**5.19**	**5.20**	19.46	**2.37**	**2.37**	2.60	**11.84**	**11.84**	14.07	2350
	1200.44	**5.54**	**5.55**	18.97	**2.05**	**2.05**	2.93	**12.78**	**12.78**	15.71	2350
	1601.58	**3.00**	**3.00**	13.67	**2.88**	**2.88**	6.80	**16.53**	**16.53**	35.41	2350
	2003.24	**3.32**	**3.31**	14.93	**1.86**	**1.86**	1.79	**11.96**	**11.96**	12.66	2350

**Table 6:** Experiment evaluation results for typical case (Type III) at different scenarios.

Cases	Ref. (cm)	RMSE (cm)			Standard Deviation (cm)			Spread of Data (cm)			Sample Size
		*AltDS*	*SDS*	*SS*	*AltDS*	*SDS*	*SS*	*AltDS*	*SDS*	*SS*	
Close	25.63	18.06	**11.99**	23.95	3.32	11.96	**1.26**	31.26	**30.86**	33.79	2000
LOS	50.08	12.36	35.11	**3.59**	4.79	13.96	**3.39**	38.60	39.65	**18.06**	2000
	75.44	12.31	16.15	**6.96**	**2.49**	3.00	4.80	**14.13**	17.88	30.31	2000
	103.90	6.67	12.31	**5.79**	**2.41**	2.49	4.05	16.57	**15.42**	25.80	2000
	125.18	**5.07**	12.21	9.40	2.17	**1.89**	2.89	**13.13**	13.25	16.65	2000
	150.30	**3.48**	8.23	8.76	2.74	**2.51**	4.74	16.38	**13.13**	29.25	2000
	175.27	**3.43**	8.44	12.44	3.39	**3.20**	3.67	**14.32**	14.54	15.89	2000
	200.77	**4.78**	9.88	6.90	**2.34**	2.41	4.01	14.44	**14.42**	22.75	2000
LOS	399.82	**1.50**	8.01	14.31	**1.46**	**1.45**	1.74	10.79	**9.97**	12.37	2450
(Hall)	806.70	**2.04**	5.79	12.78	1.92	**1.87**	4.87	**12.53**	13.84	26.27	2450
	1206.20	**2.09**	7.13	16.62	1.55	**1.53**	1.73	**13.25**	13.37	13.37	2450
	1600.20	**3.69**	4.96	17.99	**1.55**	1.57	1.84	11.28	**10.44**	16.18	2450
	2002.00	**2.93**	5.13	10.45	**2.74**	2.89	9.28	**26.34**	**22.40**	48.48	2450
Multipath	402.12	5.53	**2.12**	17.32	**1.58**	1.80	2.68	13.55	**12.13**	17.12	2350
(Corridor)	802.05	5.03	**3.12**	17.55	**1.93**	1.98	2.12	10.88	**10.20**	13.84	2350
	1200.44	4.97	**2.52**	13.86	**1.87**	2.51	4.21	**13.50**	16.65	23.84	2350
	1601.58	**3.20**	7.04	12.18	**3.02**	3.55	5.93	**18.38**	23.80	35.88	2350
	2003.24	**6.43**	10.76	8.84	**3.00**	4.83	8.84	**17.69**	26.97	40.10	2350

**Table 7:** Experimental evaluation results for variable reply times at fixed reference distance (5.494 m).

	RMSE (ms)	RMSE (cm)			Reply Time (ms)		
No. of Trials	Reply Time	Distance Error			Mean		Sample Size
	($t_{\mathrm{replyA}}-t_{\mathrm{replyB}}$)	AltDS	SDS	SS	$t_{\mathrm{replyA}}$	$t_{\mathrm{replyB}}$	
1.	0.0003	**3.44**	**3.44**	18.40	**0.4909**	**0.4913**	2850
2.	0.24	2.74	**2.27**	15.16	0.64	**0.40**	2966
3.	0.74	**3.51**	8.87	15.76	1.14	**0.40**	2435
4.	1.24	**3.63**	15.58	15.145	1.64	**0.40**	2496
5.	1.74	**3.50**	22.27	14.56	2.14	**0.40**	2523
6.	2.24	**3.72**	28.60	14.51	2.64	**0.40**	2320
7.	2.74	**3.50**	34.33	13.97	3.14	**0.40**	2454
8.	3.24	**3.51**	39.17	13.55	3.64	**0.40**	2485
9.	3.74	**3.59**	44.40	13.40	4.14	**0.40**	2574
10.	4.24	**3.51**	50.75	13.39	4.64	**0.40**	2446

## Abbreviations

The following abbreviations are used in this manuscript:

ADS-TWR	Asymmetric Double-Sided Two-Way Ranging
AltDS-TWR	Alternative Double-Sided Two-Way Ranging
DS-TWR	Double-Sided Two-Way Ranging
GPS	Global Positioning System
IMU	Inertial Measurement Unit
LOS	Line of Sight
MAC	Medium Access Control layer
NLOS	Nonline of Sight
PATD	Preamble Accumulation Time Delay
PDS-TWR	Parallel Double-Sided Two-way Ranging
PTD	Propagation Time Delay
RTD	Receiving Time Delay
RMSE	Root Mean Square Error
SDS-TWR	Symmetric Double-Sided Two-Way Ranging
SS-TWR	Single-sided Two-Way Ranging
STD	Standard Deviation
TEEM	Time-of-Flight Error Estimation Model
TOF	Time-of-Flight
TTD	Transmission Time Delay
TWR	Two-Way Ranging
WSN	Wireless Sensor Network
